# Do the total mercury concentrations detected in fish from Czech ponds represent a risk for consumers?

**DOI:** 10.1038/s41598-021-04561-5

**Published:** 2022-01-11

**Authors:** Sehonova Pavla, Harustiakova Danka, Mikula Premysl, Medkova Denisa, Malacova Kristyna, Svobodova Zdenka

**Affiliations:** 1 Department of Animal Protection and Welfare & Veterinary Public Health, Faculty of Veterinary Hygiene and Ecology, University of Veterinary Sciences, Brno, Czech Republic; 2 RECETOX, Faculty of Science, Masaryk University, Brno, Czech Republic; 3 Department of Zoology, Fisheries, Hydrobiology and Apiculture, Faculty of Agrisciences, Mendel University in Brno, Brno, Czech Republic

**Keywords:** Ecology, Environmental sciences

## Abstract

Mercury is one of the important pollutants of the environment. Therefore, it’s necessary to monitor quantity of mercury especially in aquatic ecosystems. The main goal of the presented study was to compare the content of total mercury in tissues of fish coming from the Czech Republic, an important carp exporter, with focus on comparison of mercury content between 3 different ponds, its comparison between different fish species and between different tissues of the same species, and estimation whether the mercury content in tissues meets the limit given in the Commission Regulation (EC) No. 1881/2006 or not. Total mercury concentration was measured in 90 fish specimen sampled from three ponds (Velky Kocelovicky, Mysliv and Zehunsky) in autumn 2018. The values of total mercury in fish tissues was measured by atomic absorption spectrometry. The content of total mercury in the tissues decreased as follows: muscle > liver > gonads > scales. The highest average content of total mercury in muscle was 0.1517 ± 0.0176 mg/kg coming from pike caught in Velky Kocelovicky pond. In contrast, the lowest average content of total mercury in muscle 0.0036 ± 0.0003 mg/kg was found in carp tissue coming from the locality of Zehunsky pond. We confirmed that the predatory fish are more exposed to mercury than non-predatory fish. None of the monitored localities exceeded the set regulatory limit. Thus, our study shows that fish coming from these ponds are safe in terms of total mercury content.

## Introduction

The aquatic environment contains a high amount of contaminants that can pose a risk to the human population. Mercury (Hg) is one of the most widespread and ubiquitous contaminants in aquatic ecosystems, mainly due to its global distribution through multiple pathways (air, water, and food)^1^. Mercury gets into the aquatic environment naturally or due to human activities^2–5^. The most important sources of mercury in the aquatic environment are erosion, emissions from industry, mining and combustion of fossil fuels^6–9^. Mercury has the ability to bioaccumulate, its concentration increases in the food chain, so a higher content of mercury is found in predatory fish^2,10–13^. The concentration of mercury in fish depends mainly on the concentration of mercury in the aquatic environment^14^ where, due to microorganisms contained in the bottom sediment, inorganic mercury is converted into a more available organic form called methyl mercury (MeHg) which enters the body of the fish through the food chain^12,15^. Mercury concentrations are also affected by the age and weight of fish^16,17^.

As a result, the accumulation of methyl mercury in aquatic organisms poses a risk not only for humans but also for wildlife^13,18^. In order to prevent risk connected to environmental mercury exposure, Minamata convention on mercury was signed by more than 120 countries including the Czech Republic and came into force in 2017. As a result, since 2020 the manufacture, import and export of mercury-added products, with certain exceptions, has no longer been allowed^19^.

Aquaculture has been historically a significant activity in the Czech Republic with common carp being the most dominant species. In 2017, the aquaculture production of the Czech Republic was 21,685 tons, out of these 85.1% was common carp^20^. Main part of the domestic production is intended for export^21^. Surface waters represent a reservoir of numerous environmental contaminants. Mercury and other heavy metals are able to accumulate in aquatic organisms, especially fish. Since the Czech Republic is a landlocked country having no access to ocean, freshwater aquaculture is of a high importance there. Fish pond farming, a traditional form of aquaculture in the Czech Republic, is considered as its national heritage^22^.

Human exposure to mercury occurs mainly through the consumption of aquatic animals, especially fish, which come from a contaminated environment and are consumed frequently and for a long time^23^. Fish form an integral part of the diet of a large number of people, so it is necessary to monitor the mercury content, as the highest concentrations of mercury can be found in the muscle of fish^14,24^. The mercury content in fish muscle is regulated by Commission Regulation (EC) No. 1881/2006 setting maximum levels for certain contaminants in foodstuffs. The regulation distinguishes between two groups of fish. While the maximum level of 0.5 mg/kg (wet weight) has been set for freshwater fish with the exception of pike (*Esox lucius*), eel (*Anguilla* spp.) and sturgeon (*Acipenser* spp.), up to 1 mg/kg (wet weight) of mercury can be detected in marine fish and pike, eel and sturgeon. The limits in the regulation are expressed as total mercury (THg). However, MeHg is primarily responsible for bioaccumulation in the muscle tissue of fish with a MeHg fraction of 83–90% of the-total mercury concentration^25^. Therefore, World Health Organization has set provisional tolerable weekly intake for MeHg to be 1.6 µg/ kg human body weight/week^26^.

The aim of the present paper was to analyze and present the results of research on mercury content in tissues of fish coming from the Czech Republic, an important carp exporter, with focus on comparison of mercury content between 3 different ponds, comparison of mercury content between different fish species and between different tissues of the same species, and estimation whether the mercury content in tissues meets the limit given in the Commission Regulation (EC) No. 1881/2006 or not.

## Materials and methods

**Figure 1:**
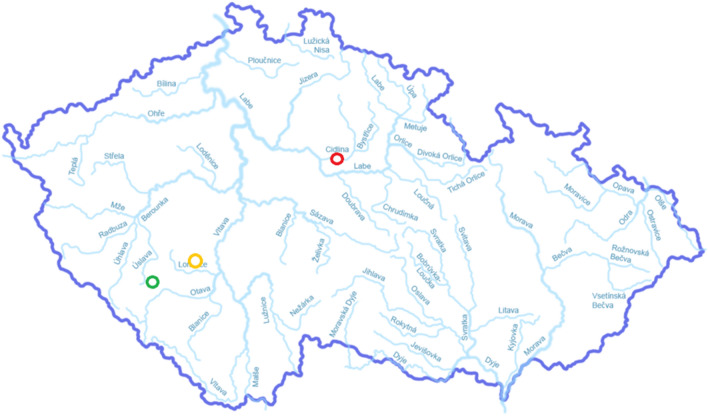
Location of the sampling sites: red—Zehunsky pond, yellow—Mysliv pond, green—Velky Kocelovicky pond.

To evaluate the content of THg, 3 ponds were selected: Zehunsky, Mysliv and Velky Kocelovicky. The location of the ponds is shown in Fig. 1. The Zehunsky pond (Chlumec nad Cidlinou fishing) has an area of approximately 258 ha. Mysliv pond (Klatovy fishing) has an area of 60 ha. The Velky Kocelovicky pond (Lnare fishing) has an area of 33 ha. The fish stock in these ponds is composed primarily of common carp (*Cyprinus carpio*) and secondary species of predatory fish. These fish are represented by catfish (*Silurus glanis*), pike (*Esox lucius*) and zander (*Sander lucioperca*). These three ponds were selected because of their high yield of fish intended for human consumption. Mysliv pond is located in West Bohemia and Velky Kocelovicky in the South Bohemian region, which is considered the most important terms of aquaculture. The Zehunsky pond is located in the Central Bohemian region and is the largest pond there. The fish were caught during the autumn season together with fish for trading.

The fish were killed by a blunt blow to the head, followed by an overcut of the gill arch to bleed. The fish were weighed and the total body length was measured. The characteristics of the analyzed fish are given in Table 1. Muscle, liver, gonad and scale samples were taken from these fish during autopsy. The samples were stored at − 20 °C until analysis. Scales were also samples to determine the age of the fish.

**Table 1:** Age, total body length and body weight of fish (mean ± standard error of mean) caught at three ponds: Velky Kocelovicky (VK), Mysliv (M) and Zehunsky (Z).

Pond	Species	N	Age (years)	Total body length (cm)	Weight (g)
VK	Carp	10	4.1 ± 0.2	51.3 ± 0.8	2981.5 ± 66.5
VK	Pike	10	3.3 ± 0.2	58.9 ± 2.5	1327.0 ± 174.7
VK	Zander	10	3.0 ± 0.0	42.3 ± 0.3	722.0 ± 26.0
M	Carp	10	3.3 ± 0.2	38.3 ± 0.4	1250.5 ± 57.9
M	Pike	10	3.4 ± 0.2	59.0 ± 2.5	1537.5 ± 153.8
M	Zander	10	3.4 ± 0.2	49.3 ± 0.7	1292.0 ± 42.9
Z	Carp	10	4.2 ± 0.2	46.7 ± 0.6	2035.5 ± 87.2
Z	Pike	10	3.5 ± 0.2	57.1 ± 2.0	1459.0 ± 152.5
Z	Catfish	10		60.0 ± 3.1	2027.0 ± 390.3

The age of the fish was determined by visual inspection of scales with a help of binocular magnification instrument Meoflex RI 21P (Meopta, Czech Republic) with the exception of catfish, where the age was not determined due to the absence of scales.

The content of THg was determined on a single-atomic absorption spectrometer AMA 254 (Altec Ltd., Czech Republic), which allows determination of THg without sample pre-treatment. The accuracy of the results was verified using standard reference material (CRM No. 13 HUMAN HAIR and BCR-CRM 464, Tuna, IRMM, Belgium). The limit of quantification (LOQ) was 0.001 mg/kg. The THg is expressed in mg/kg fresh weight of analyzed tissues.

The THg liver/muscle ratio is the ratio of liver to muscle Hg concentrations and is calculated as follows: [THg in liver (mg/kg)/THg in muscle (mg/kg)]. THg liver/muscle ratio was calculated from the arithmetic mean.

Hazard index was calculated on the basis of the Kannan et al.^27^ method. The hazard index (HI) for mercury is ratio dose (D) to the upper level of daily mercury intake during a lifetime estimated without toxic effects (RfD). The formula for this calculation is as follows: HI = D/RfD. The estimated dose (D) can be calculated as D = C × I/W × 1000 where C = concentration of mercury in fish (µg/g), I = ingestion rate of fish (g/day), W = average body weight (70 kg). RfD value for mercury is 1 × 10^–4^ mg/kg/day^28^. In the Czech Republic, annual consumption of freshwater fish is 1.3 kg per person^29^. If the hazard index is less than 1, the mercury exposure could be regarded as unlikely to lead to adverse health effects.

### Statistical analysis

A total of 10 individuals of each fish species from each pond were analyzed. The age of fish between ponds was compared by one-way ANOVA (carp, pike) and t-test (zander). The mercury content in muscle, liver, gonads and scales of each fish was measured. Factorial ANOVA followed by the Tuckey post hoc test were used to assess the effect of sampling site, fish species and fish tissue on THg. The same test was used to analyse differences in mercury content in gonads between males and females taking into account sampling site and fish species.

P < 0.05 was considered significant in all tests. Statistical analyses were performed using Statistica, version 13 (TIBCO Software Inc.).

### Ethical statement

All the methods used in this study followed relevant guidelines and regulations, in particular Act No. 246/1992 Coll., on the Protection of Animals against Cruelty, as amended and Decree No. 419/2012 Coll., on the Protection, Breeding and use of Experimental Animals, as amended. Moreover, the competent authority (Ethical Committee for Protection of Animals in Research of the University of Veterinary Sciences Brno) approved the fish sampling and protocols of the present study and reporting herein follows the recommendations in the ARRIVE guidelines.

## Results

The age of the analyzed fish ranged from 3.0 to 4.2 years. Higher age of carp was found in the Zehunsky and Velky Kocelovicky pond, lower in the Mysliv pond (Table 1). The difference was significant (ANOVA, F(2,26) = 6.408, P = 0.005). The age of pike did not differ significantly between ponds (ANOVA, F(2,27) = 0.300, P = 0.743), the same applies to zander (t-test, t(18) = 1.809, P = 0.087).

Fish used for the determination of THg were caught in the ponds Velky Kocelovicky, Zehunsky and Mysliv.

THg in fish differed significantly between studied ponds, fish species and tissue (factorial ANOVA, effect of studied pond: F(2,323) = 20.468, P < 0.001; effect of fish species: F(3,323) = 41.243, P < 0.001; effect of tissue: F(3,323) = 120,591, P < 0.001; effect of interaction of all three predictors: F(18,323) = 5.815, P < 0.001).

**Figure 2:**
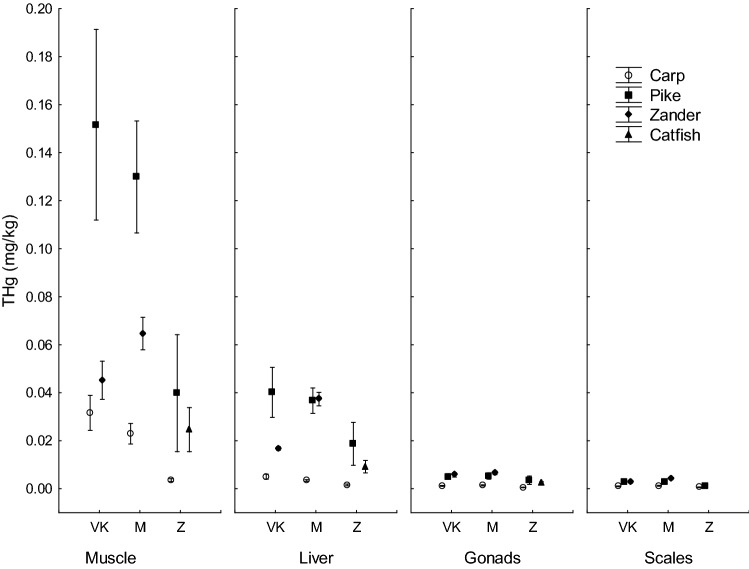
Total mercury content (mg/kg wet weight) in fish tissue of carp, pike, zander and catfich caught in three sampling sites: Velky Kocelovicky (VK), Mysliv (M) and Zehunsky (Z). Vertical bars denote 0.95 confidence intervals.

The highest content of THg in fish from Velky Kocelovicky pond was observed in the muscle of pike (0.1517 ± 0.0176 mg/kg), the lowest content was determined in samples of scales and gonads of carp (0.0010 ± 0.0001 and 0.0012 ± 0.0001 mg/kg). In the Mysliv pond, the highest content of mercury was measured in the muscle of pike (0.1299 ± 0.0103 mg/kg), whereas the lowest values were observed in the scales and gonads of common carp (0.0013 ± 0.0001 and 0.0014 ± 0.0001 mg/kg). The situation was the same in Zehunsky pond; the highest content of mercury was measured in the muscle of pike (0.0398 ± 0.0108 mg/kg) and the lowest values were determined in the gonads of common carp (0.0006 ± 0.0000 mg/kg) and in the scales of carp and pike (0.0007 ± 0.0001 and 0.0012 ± 0.0001 mg/kg) (Table 2, Fig. 2).

**Table 2:** Total mercury content THg (mg/kg wet weight; mean ± standard error of mean) in fish tissue of carp, pike, zander and catfish caught in three ponds: Velky Kocelovicky (VK), Mysliv (M) and Zehunsky (Z).

Pond	Species	N	THg in muscle (mg/kg)	THg in liver (mg/kg)	THg in gonads (mg/kg)	THg in scales (mg/kg)
VK	Carp	10	0.0316 ± 0.0032^A,a,*A*^	0.0050 ± 0.0004^A,a,*A*^	0.0012 ± 0.0001^A,a,*A*^	0.0010 ± 0.0001^A,a,*A*^
VK	Pike	10	0.1517 ± 0.0176^B,a,*A*^	0.0402 ± 0.0046^A,a,*B*^	0.0051 ± 0.0005^A,a,*BC*^	0.0027 ± 0.0003^A,a,*C*^
VK	Zander	10	0.0452 ± 0.0035^A,a,*A*^	0.0168 ± 0.0003^A,a,*AB*^	0.0058 ± 0.0004^A,a,*B*^	0.0028 ± 0.0002^A,a,*B*^
M	Carp	10	0.0229 ± 0.0019^A,a,*A*^	0.0034 ± 0.0002^A,a,*A*^	0.0014 ± 0.0001^A,a,*A*^	0.0013 ± 0.0001^A,a,*A*^
M	Pike	10	0.1299 ± 0.0103^B,a,*A*^	0.0367 ± 0.0023^A,a,*B*^	0.0053 ± 0.0006^A,a,*B*^	0.0030 ± 0.0002^A,a,*B*^
M	Zander	10	0.0647 ± 0.0030^C,a,*A*^	0.0373 ± 0.0012^A,a,*AB*^	0.0067 ± 0.0003^A,a,*B*^	0.0042 ± 0.0002^A,a,*B*^
Z	Carp	10	0.0036 ± 0.0003^A,a,*A*^	0.0016 ± 0.0002^A,a,*A*^	0.0006 ± 0.0000^A,a,*A*^	0.0007 ± 0.0001^A,a,*A*^
Z	Pike	10	0.0398 ± 0.0108^A,b,*A*^	0.0187 ± 0.0039^A,a,*AB*^	0.0036 ± 0.0008^A,a,*AB*^	0.0012 ± 0.0001^A,a,*B*^
Z	Catfish	10	0.0246 ± 0.0041^A,–,*A*^	0.0092 ± 0.0012^A,–,*A*^	0.0024 ± 0.0002^A,–,*A*^	

Total mercury content in the tissue of different fish species in one pond followed by the same capital letter in the column did not differ significantly (Tuckey post hoc test in factorial ANOVA; separately for VK, M, Z).

Total mercury content in the tissue of one species in different ponds followed by the same lower-case letter in the column did not differ significantly (Tuckey post hoc test in factorial ANOVA; separately for carp, pike, zander).

Total mercury content in different tissues followed by the same capital letter in italics in the row did not differ significantly (Tuckey post hoc test in factorial ANOVA).

THg content in tissues decreased in the order: muscle > liver > gonads > scales. Differences between tissues were significant (P < 0.05) for pike, zander and catfish in all ponds. For carp, the THg in different tissues did not differ significantly (P > 0.05) in any pond (Table 2).

In the locality Velky Kocelovicky pond, a statistically significant difference (P < 0.05) was found in the content of THg in the muscle of pike and the other two species (common carp, zander). No significant difference (P > 0.05) was found in THg content in the muscle of common carp and zander. Considering the Mysliv pond, a statistically significant difference (P < 0.05) was found between all three species, being the highest in pike and the lowest in carp. In the Zehunsky pond, no significant interspecific differences (P > 0.05) were found in the mercury content in fish muscle (Table 2).

The THg content in the muscle of carp varied from 0.0036 ± 0.0003 mg/kg in Zehunsky pond to 0.0316 ± 0.0032 mg/kg in Velky Kocelovicky and did not differ significantly between the three ponds (P > 0.05). Similarly, in zander the mercury content in muscle did not differ (P > 0.05) between Velky Kocelovicky (0.0452 ± 0.0035 mg/kg) and Mysliv (0.0647 ± 0.0030 mg/kg). The situation was different for pike, where the mercury content in the muscle ranged from 0.0398 ± 0.0108 mg/kg in Zehunsky to 0.1517 ± 0.0176 mg/kg in Velky Kocelovicky and differed significantly (P < 0.05) (Table 2).

The mercury content in the liver of fish ranged from 0.0016 ± 0.0002 mg/kg (carp, Zehunsky pond) to 0.0402 ± 0.0046 mg/kg (pike, Velky Kocelovicky) and did not differ significantly either between species or sampling sites (Table 2).

In scales, the lowest mercury content was 0.0007 ± 0.0001 mg/kg (carp, Zehunsky) and the highest 0.0042 ± 0.0002 mg/kg (zander, Mysliv). The mercury content in scales did not differ significantly either between fish species or sampling sites (Table 2).

The mercury content in the gonads ranged from 0.0006 ± 0.0000 mg/kg (carp, Zehunsky pond) to 0.0067 ± 0.0003 mg/kg (zander, Mysliv) and did not differ significantly either between species or sampling sites (Table 2).

**Figure 3:**
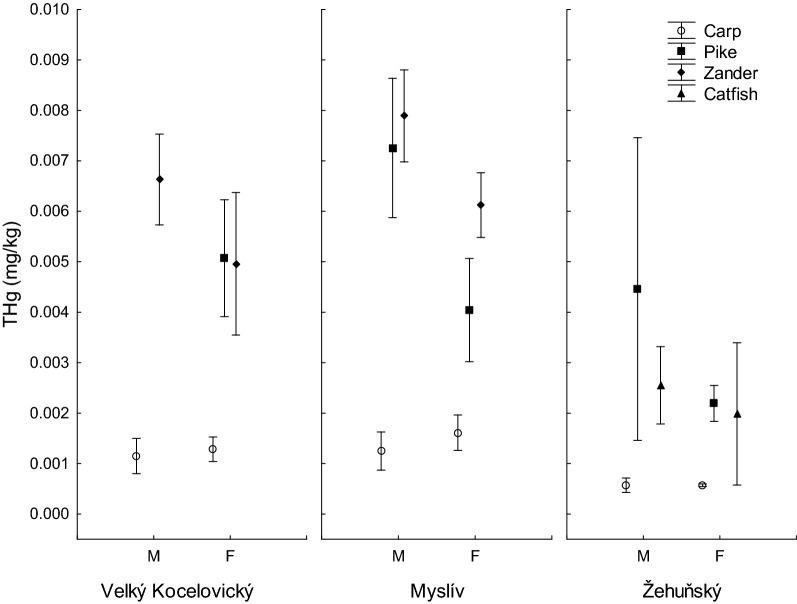
Total mercury content (mg/kg wet weight) in gonads of male (M) and female (F) carp, pike, zander and catfich caught in three sampling sites. Vertical bars denote 0.95 confidence intervals.

A detailed analysis of the mercury content in the gonads of male and female fish found values from 0.0006 ± 0.0001 mg/kg and 0.0006 ± 0.0000 mg/kg, respectively (male and female, carp, Zehunsky pond) to 0.0079 ± 0.0002 (male, zander, Mysliv) (Table 3, Fig. 3). Factorial ANOVA used to identify differences in mercury content in gonads between males and females and taking into account sampling site and fish species did not find significant differences between males and females (effect of gender in factorial ANOVA: F(1,77) = 2.839, P = 0.096). Despite this, the Tuckey post hoc test revealed differences in the mercury content in the gonads of males and females of pike from the Mysliv pond; values were higher in males (Table 3).

**Table 3:** Total mercury content THg (mg/kg wet weight; mean ± standard error of mean) in the gonads of male and female carp, pike, zander and catfish caught in three ponds: Velky Kocelovicky (VK), Mysliv (M) and Zehunsky (Z).

Pond	Species	Male		Female	
		N	THg in gonads (mg/kg)	N	THg in gonads (mg/kg)
VK	Carp	6	0.0012 ± 0.0001^*A*^	4	0.0013 ± 0.0001^*A*^
VK	Pike			10	0.0051 ± 0.0005^*–*^
VK	Zander	5	0.0066 ± 0.0003^*A*^	5	0.0050 ± 0.0005^*A*^
M	Carp	5	0.0012 ± 0.0001^*A*^	5	0.0016 ± 0.0001^*A*^
M	Pike	4	0.0073 ± 0.0004^*A*^	6	0.0040 ± 0.0004^*B*^
M	Zander	3	0.0079 ± 0.0002^*A*^	7	0.0061 ± 0.0003^*A*^
Z	Carp	7	0.0006 ± 0.0001^*A*^	3	0.0006 ± 0.0000^*A*^
Z	Pike	6	0.0045 ± 0.0012^*A*^	4	0.0022 ± 0.0001^*A*^
Z	Catfish	7	0.0026 ± 0.0003^*A*^	3	0.0020 ± 0.0003^*A*^

Total mercury content in the gonads of males and females followed by the same capital letter in italics in the row did not differ significantly (Tuckey post hoc test in factorial ANOVA).

THg liver/muscle ratios was calculated for fish from individual ponds. Ratio higher than 1 means very contaminated location. If ratio is lower than 1 it is insignificant contamination. As these values are far below 1, they indicate that the fish we monitored came from uncontaminated sites. The highest THg liver/muscle ratio was calculated for catfish from Mysliv pond (Table 4).

**Table 4:** Total mercury (THg) liver/muscle (L/M) ratios and Hazard index (HI) in carp, pike, zander and catfish caught in three ponds: Velky Kocelovicky (VK), Zehunsky (Z) and Mysliv (M).

	VK		Z		M	
	L/M ratio	HI	L/M ratio	HI	L/M ratio	HI
Carp	0.217	0.016	0.500	0.002	0.013	0.012
Pike	0.263	0.078	0.475	0.021	0.285	0.067
Zander	0.378	0.023			0.569	0.033
Catfish			0.360	0.013		

Where data are missing – indicated species was not caught in tht particular pond.

Hazard index (HI) was calculated for every fish species from three ponds. The highest hazard index was found for pike from the pond Velky Kocelovicky 0.078 and subsequently for pike from the pond Mysliv 0.067. The lowest HI was found in carp 0.002 from pond Zehunsky (Table 4).

## Discussion

Mercury content in fish depends on several factors. The first one and one of the most important factors represent the environmental contamination. Generally higher THg concentrations can be expected in fish from areas with a high industrial load, in the proximity of big cities, especially downstream from possible sources of contamination^30–32^. From this viewpoint, low THg concentrations detected in fish from our study are not surprising, as all the 3 breeding ponds were located in countryside far from any industrial plants. Comparable THg concentrations were previously found in common carp cultured in 10 other Czech uncontaminated fishponds^24^, two different farms/ponds in Austria^13^, Shadegan International Wetland located in Iran^23^ and also in carp from other freshwater systems^33–35^. For instance, Vicarova et al.^35^ monitored the contamination of three water reservoirs in the Bohemian-Moravian Highlands. While THg concentrations detected in carp muscle ranged from 0.0189 to 0.0724 mg/kg, up to 0.0189 mg/kg of mercury was found in liver^35^. These values correspond well to results of our study.

Due to concerns about possible adverse effects of mercury on fish and also humans consuming them, extensive legislative measures regulating industrial use of mercury have been adopted. All of these measures can potentially lead to the decrease in THg concentrations detected in fish as well as other aquatic organisms. In fact, this was already proven in our previous study investigating spatial and temporal trends in contamination of the Czech part of Elbe River^30^. Overall long-term decline in THg concentrations of at least 20% during 1965–2012 was also observed in Swedish freshwater fish^36^.

The age of fish is another factor affecting THg concentrations detected in fish tissues as it is expected that the bioaccumulation of this heavy metal proceeds throughout the whole life of fish. A significant positive correlation between the age of fish and the THg concentrations detected in their muscles was previously found in chub (*Leuciscus cephalus*) from Morava River basin^37^. Age-related differences in THg concentrations detected in fish tissues were also reported by Dusek et al.^38^ and Marsalek et al.^39^. Although we intended to investigate the fish of similar age, statistical analysis of our data revealed that there was a small but still significant difference in the age of carp from Mysliv pond compared to those caught in Velky Kocelovicky or Zehunsky ponds. This should be taken into account when interpreting data.

Feeding habits of fish affect bioaccumulation of mercury as well. Compared to herbivores, limnivores or omnivores, relatively higher mercury levels were previously detected in predatory species of fish representing a higher trophic level^2,10–12,40^. This is in the full agreement with our study, as generally lower concentrations of mercury were detected in the common carp (omnivorous fish), compared to 3 other carnivorous species (pike, zander, catfish). The significant differences in THg concentrations in fish muscle were revealed between common carp and pike sampled from Velky Kocelovicky and Mysliv pond.

According to some authors, fish sex can also potentially affect bioaccumulation of mercury. By examining possible relationships between the sex of perch (*Perca fluviatilis*) and mercury accumulation in their gonads, Jankovska et al.^41^ observed that male gonads (milt) can accumulate higher levels of mercury than do female spawn, probably due to their different compositions^41^. Other authors focused on differences in THg concentrations detected in muscle tissue between both female and male fish. While female European catfish (*Silurus glanis*) from Italian rivers displayed higher concentrations of mercury compared to male specimens^32^, no significant differences between both sexes were revealed in fish from Ebro River (Spain)^42^. In our study, THg concentrations detected in fish gonads did not significantly differ between both sexes of fish, excepting the pike from Mysliv pond, where significantly higher THg concentrations were observed in male fish compared to those found in females.

A distribution of mercury in fish tissues has been considered as a good indicator of environmental contamination by this heavy metal, since it was reported, that the deposition of mercury in fish differs between uncontaminated and heavily contaminated sites. While liver represents the target organ for mercury accumulation in fish from highly contaminated localities, in the case of lightly contaminated (or uncontaminated) sites, the majority of mercury is being deposited into fish muscle^43,44^. THg concentrations which we detected in various fish tissues decreased in the following order: muscle > liver > gonads > scales. This is in a good agreement with previous study by Kensova et al.^45^. Similar results of tissue distribution of mercury with more contaminant detected in carp muscle compared to liver were also obtained by Zhang et al.^14^. However, these authors did not measure THg concentrations in gonads and scales as we did, but chose intestine and gills instead^14^.

One of the aims of our study was to compare THg concentrations detected in fish from the three ponds to limits on Hg content stated by current food legislation, especially the Commission Regulation 1881/2006 setting maximum levels for certain contaminants in foodstuffs. According to this legal act, up to 0.5 mg/kg of mercury can be present in muscle of fish and fishery products marketed in the EU, unless they belong to certain predatory species listed in the section of 3.3.2 of the Annex I of this regulation. Since pike has been listed in this section, a maximum level of mercury of 1.0 mg/kg is relevant for this species^46^. Regardless of that, THg concentrations detected in all three ponds (Mysliv, Zehunsky, Velky Kocelovicky) were substantially lower than limit values. This suggests that the consumption of fish from the ponds does not represent health risks for consumers.

Not only on the basis of the limits set by legislation, but also the results of the hazard index show that fish come from safe locations in term of Hg contamination. The highest hazard index was found in predatory fish especially in pike (0.078 Velky Kocelovicky pond; 0.067 Mysliv pond). The lowest hazard index as expected was calculated in carp (0.002 Zehunsky pond, 0.012 Mysliv pond; 0.016 Velky Kocelovicky pond). It follows that the values of the hazard index are far from 1 and therefore the consumption of fish from these ponds is safe. Novotna et al.^30^ set the values of the hazard index for fish from the river Elbe, which flows through the Czech Republic. Their hazard index results for fish from this locality are also below 1, therefore no negative impact on the health of people consuming fish from this river is expected.

## Conclusion

Mercury content in fish did not exceed legislative limits in any of the three monitored production ponds. Our study shows that farmed fish production in ponds are safe for human consumption in terms of THg concentration. In the Czech Republic, the most commonly consumed fish in open waters is common carp. For common carp, the measured values of THg were at a very low level. We also confirmed in the study that a higher content of mercury content is found in predatory fish, which in our case were pike, zander and catfish.
